# Combination therapy for HCC: from CRISPR screening to the design of clinical therapies

**DOI:** 10.1038/s41392-021-00775-1

**Published:** 2021-10-04

**Authors:** Yunguang Wang, Ling Kui, Guangchuan Wang

**Affiliations:** 1 Department of Critical Care Medicine, Affiliated Hangzhou First People’s Hospital, Zhejiang University School of Medicine, Hangzhou, China; 2 State Key Laboratory of Molecular Biology, Shanghai Institute of Biochemistry and Cell Biology, Center for Excellence in Molecular Cell Science, Chinese Academy of Sciences, Shanghai, China; 3 Shenzhen Qianhai Shekou Free Trade Zone Hospital, Shenzhen, China; 4 Dana-Farber Cancer Institute, Harvard Medical School, Brookline, MA United States

**Keywords:** Drug development, Drug development, Genetic techniques

CRISPR screening has been broadly used to discover new therapy targets, but these finding’ successful clinical transition is still limited. In a recent study published in Nature, Jin et al.^1^ adopted CRISPR screening to dissect the resistance of hepatocellular carcinoma to lenvatinib, discovering the synergistic effect of EGFR inhibitor (gefitinib) and lenvatinib (multi-tyrosine kinase inhibitor, muti-TKI) combination therapy with encouraging clinical benefits.

Liver cancer is still a major threat to global health, ranking as the fourth most common cause of cancer-related death and the second most lethal tumor, with a 5-year survival rate of 18%. Unfortunately, the global incidence of liver cancer is still growing, with estimated cases of >1 million by 2025^2^. Hepatocellular carcinoma (HCC) is the most common type of primary liver cancer. Despite the large armamentarium of therapies that have been used to treat HCC, including surgery, ablation therapy, embolization, chemotherapy, and radiation therapy, the majority of late-stage HCC patients succumb to the disease. In advanced stages, only systemic therapy is applicable. For decades, sorafenib, an oral multi-TKI, was the only approved systemic therapy for HCC. Recently, TKIs and immune-checkpoint inhibitors (ICIs) have changed conventional therapies for HCC. Among them, lenvatinib showed high efficacy (median overall survival of 13.6 months for lenvatinib vs 12.3 months for sorafenib) in a global open-label randomized phase III study and was approved as the new first-line for advanced-stage HCC^3^. This result puts lenvatinib as one of the best single therapies for HCC. However, the overall response rate for lenvatinib is only 24.1%^3^, highlighting the need for developing combination therapies. The scenario was supported by promising results observed in the combination of ICIs and TKIs or anti-angiogenic therapies. For example, the combination of PD-L1 antibody (atezolizumab) and VEGF inhibitor (bevacizumab) has demonstrated striking superiority in first-line treatment, opening a new standard of care for HCC. However, the overall survival and response rate for patients with advanced HCC is still very low, reflecting the need to explore new combination therapies.

A recent milestone study by Jin et al. reports the use of synthetic lethal CRISPR screen in discovering the synergistic effects of EGFR inhibitor and lenvatinib combination in HCC, achieving an approximately 50% of clinical responses in patients with advanced HCC who were unresponsive to lenvatinib^1^. In this study, the authors used SNU499, a lenvatinib-resistant HCC cell line, to carry out synthetic lethal CRISPR screens, and discovered the knockout of EGFR under lenvatinib treatment is lethal to EGFR-expressing HCC cells. Considering the fact that most liver cancers express high levels of EGFR, they explored the therapeutic potentials of combinating EGFR targeting gefitinib and lenvatinib in HCC. According to the in vitro and in vivo data, the combination of lenvatinib and gefitinib had potent inhibition of tumor growth and their synergistic effect seems only applied to EGFR^high^ cells. Nevertherless, another seemingly similar EGFR inhibitor plus multi-TKI combination pair, erlotinib and sorafenib, failed to yield significant clinical benefit. Exploring the underlying reasons, they noticed although both sorafenib and lenvatinib are multi-TKIs, only lenvatinib inhibits FGFR signaling which is essential for the combination therapy, and the combination of FGFR inhibitor and EGFR inhibitor had similar synergistic effects against liver cancer. Further mechanistic studies guided by the CRISPR screening data revealed that the feedback activation of the EGFR-PAK2-ERK5 pathway after lenvatinib treatment contributes to the resistance of HCC to lenvatinib. Therefore, the addition of the EGFR inhibitor, gefinitib, abolishes or at least alleviates the resistance of HCC to lenvatinib by suppressing this pathway. Based on the above mentioned results, they initiated a clinical trial to evaluate the therapeutic potentials of lenvatinib and gefitinib combination in patients with unresectable, lenvatinib-resistant, EGFR^high^ HCC. Although still early, this combination therapy showed encouraging results in patients with advanced HCC who are not responsive to the treatments of levatinib or lenvatinib plus PD-1 antibody. According to the mRECIST criteria, 4 of 12 cases showed confirmed partial response, 4 showed stable disease, and 4 showed progression disease. Notably, according to their MRI data, the responders already showed sharp tumor burden reduction and serum AFP decrease within 4 weeks of the combination therapy. Even promising, it might still be early to draw firm conclusions, and we are eager to see the long-term survival outcome.

It has to be mentioned that the vast majority of patients with advanced HCC still had a very poor prognosis due to primary or acquired resistance to systemic therapies. This seminal study has demonstrated that, by designing appropriate CRISPR screens, we can systematically dissect the pathways and mechanisms of the drug resistance and the identified key regulators especially the synergistic targets that may hold great potentials for new combination therapies (Fig. 1). Notably, this is not their first effort to identify potential synergistic targets for combination therapy with CRISPR screening. At 2019, Prof. Wenxin Qin’s group and Prof. Rene Bernards’s group already performed CRISPR screening on HCC vulnerabilities and identified the combined inhibition of CDC7 and mTOR as a “one-two punch” therapy for liver cancer with TP53 mutation, reducing tumor relapse by alleviating both the cell-autonomous and non-cell-autonomous attributes of senescent cells^4^. This time they further pushed the study from “bench to bed”, substantiating the power of high-throughput screening in guiding the design of clinical combination therapy.

**Fig. 1:**
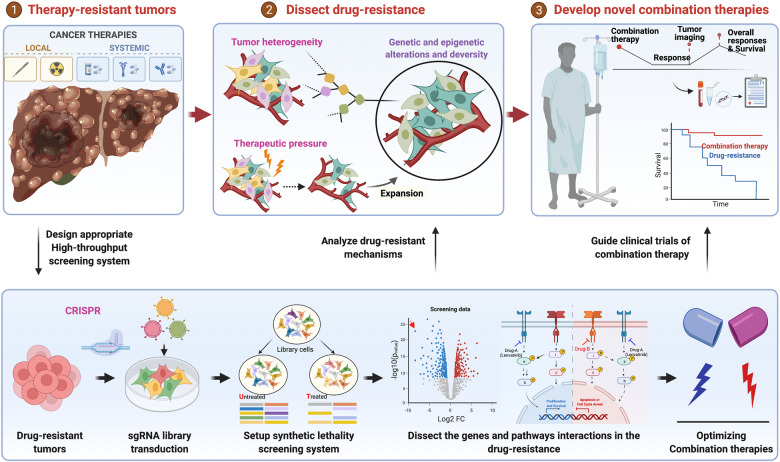
Rational design of novel clinical trials through CRISPR screening. Majority of the HCC patients receiving systemic therapies either show primary resistance or develop acquired resistance later. With high-throughput CRISPR screening technology, appropriate clinical issue-targeting genetic screens can be designed to elucidate the pathways and mechanisms involved in the drug resistance of HCC and discover new synergistic therapy targets. And the benchtop identified single or combination therapy targets that can be used to guide small scale clinical trials. The above scenario may provide a new avenue of developing more effective combination therapies. (The schematic was created with BioRender.com)

Nowadays, high-throughput CRISPR screening has been broadly used to discover new single/combination therapy and immune-oncology targets. For example, Wei et al. performed a genome-wide CRISPR/Cas9 screening and identified phosphoglycerate dehydrogenase as a critical driver for sorafenib resistance.^5^ Besides, we also have performed direct in vivo CRISPR screenings under checkpoint immunotherapy settings and identified several immunotherapy-relevant targets. Nevertheless, this is the first study that successfully paves the way from a benchtop CRISPR screen to a bedside clinical trial.

Furthermore, the complex genetic interactions under cancer-immune settings are growing to be analyzed by combinatorial CRISPR screening. Further connecting the abovementioned screening with clinical issues will improve the availability of this powerful platform in analyzing the pathway dispensability and interactions in immunotherapy resistance. In view of the prevalence of drug resistance and CRISPR screening, we expect this seminal work can spark this “hot field” to “burn out” more malignant tumors with new and more effective combination therapies.

## References

[1] Jin H (2021). EGFR activation limits the response of liver cancer to lenvatinib. Nature.

[2] Llovet JM (2021). Hepatocellular carcinoma. Nat. Rev. Dis. Prim..

[3] Kudo M (2018). Lenvatinib versus sorafenib in first-line treatment of patients with unresectable hepatocellular carcinoma: a randomised phase 3 non-inferiority trial. Lancet.

[4] Wang C (2019). Inducing and exploiting vulnerabilities for the treatment of liver cancer. Nature.

[5] Wei L (2019). Genome-wide CRISPR/Cas9 library screening identified PHGDH as a critical driver for Sorafenib resistance in HCC. Nat. Commun..

